# Task sharing and performance of Caesarean section by the Assistant Medical Officers in Tanzania: What have we learned?

**DOI:** 10.24248/eahrj.v4i2.638

**Published:** 2020-11-26

**Authors:** Nathanael Sirili, Lilian Mselle, Amani Anaeli, Siriel Massawe

**Affiliations:** a Department of Development Studies, Muhimbili University of Health and Allied Sciences, Dar es Salaam, Tanzania; b Department of Clinical Nursing, Muhimbili University of Health and Allied Sciences, Dar es Salaam, Tanzania; c Department of Obstetrics and Gynaecology, Muhimbili University of Health and Allied Sciences, Dar es Salaam, Tanzania

## Abstract

**Background:**

Since the 1960s, Tanzania adopted the task shifting which was later termed task-sharing strategy in efforts to address the critical shortage of health workforce. However, poor maternal health indicators have remained a big challenge despite this strategy having introduced mid-level cadres (Assistant Medical Officers) capable of performing roles that otherwise were performed by doctors at the district level.

**Objective:**

To analyse lessons from the performance of Caesarean section by Assistant Medical Officers (AMOs) in Tanzania as part of the task sharing strategy.

**Methods:**

An exploratory qualitative case study was carried out where 10 key informant interviews with AMOs and 4 focused group discussions with AMO trainees were conducted in 4 selected districts and 2 AMO training schools in Tanzania. With the aid of Nvivo10 qualitative software, content analysis was performed to the gathered data.

**Results:**

Performance of the Caesarean section by the AMOs is motivated by the support from various stakeholders towards improving the performance of Caesarean section. Frustrating work environment and poor incentive system are major demotivators to the performance of the Caesarean section by the AMOs.

**Conclusions:**

More than 5 decades since the introduction of AMOs through task sharing, the performance of caesarean section by these cadres face more demotivators than the motivators. Efforts should be focused on improving the work environment and provision of appropriate incentives to the AMOs. Also, more stakeholders should be engaged to support the performance of caesarean section by the AMOs for realisation of the objectives of task sharing strategy.

## BACKGROUND

Task sharing formerly known as task shifting is the name given to the process whereby less specialised health workers takes on some of the responsibilities of more specialised workers in a cost-effective manner without sacrificing the quality of care ^1^. Task sharing may also involve the delegation of some delineated tasks to newly created cadres of health workers who receive specific competency-based training^1^. ask sharing in Tanzania dates back to 1930s when the country established non-clinician physician cadres to work as physicians due to a critical shortage of properly trained physicians. It is from this evolution that the country introduced the Assistant Clinical Officers, Clinical Officers and Assistant Medical Officers training programs in the 1960s^2^. In Tanzania se deaths with the highest maternal mortality ratio of 546 per 100,000 live births^8^. In Tanzania similar to many other countries, much focus on task sharing has been in maternal health and HIV/AIDS^3,4^. This is due to the high maternal mortality and HIV/AIDS burden in many countries that are suffering from the critical shortage of Human Resources for Health (HRH) since the 1960s^1,5,6^. However, despite the global efforts geared towards task sharing in even after the Alma Ata declaration which emphasised the need for urgent action by all governments, all health and development workers, and the world community to protect and promote the health of all people^7^; maternal deaths have remained unacceptably high. Estimates show that by 2015, the global annual maternal deaths stood at 302,0008 with 99% of these deaths occurring in low and middle-income countries. Sub-Saharan Africa (SSA) contributed to 66% of these deaths with the highest maternal mortality ratio of 546 per 100,000 live births^8^.

Tanzania is among the countries with high maternal mortality in the world^9^. Despite Tanzania making progress in addressing the Millennium Development Goal number five (MDG-5), to improve maternal health; witnessed by increasing antenatal clinic visits from 96% to 98%; and from 43% to 51% for women making at least 1 of the 4 recommended visits respectively^10^; and the reduction of maternal deaths from 578 to 432 per 100,000 live births between the 1990s and 2015^11^. However, there has been a sudden increase in maternal deaths to 556 per 100,000 deaths in 2016^10^. The latter is contrary to the increased number of women who delivered under the supervision of a skilled attendant that rose from 51% in 2011 to 64% in 2015^10^. The high maternal mortality ratio does not only questions the functioning of task sharing but also threaten the progress of Tanzania in attaining the Sustainable Development Goal Number Three (SDG-3). The SDG-3 is about improving the health and wellbeing of the people. One of its targets is to reduce the global maternal mortality ratio to less than 70 per 100,000 live births by 2030.

High maternal deaths in Tanzania is attributed to; weak health infrastructure, limited access to quality health services including Caesarean section deliveries, shortage of skilled health providers, weak referral systems and weak health management at all levels^12^.

One of the backbones for the reduction of maternal mortality is the existence of a skilled and motivated workforce that is capable to diagnose and perform Caesarean section when needed. To ensure improved access to Caesarean section services, Assistant Medical Officers (AMOs), a cadre that was introduced in 1963 as part of task shifting strategy are equipped to perform Caesarean section deliveries and perform other similar roles as those performed by the medical doctors at the district level^2^. Furthermore, on-job training by different stakeholders have been provided to the AMOs on Comprehensive Emergency Obstetric and Newborn Care (CEmONC). The CEmONC interventions include safe blood transfusion, providing oxytocin and antibiotics, performing Caesarean sections, manual removal of the placenta, assisted vaginal delivery, abortion and resuscitation of the newborn^13^.

However, despite having about 1,700 AMOs in the country as of 2010, access to Caesarean section deliveries has remained low. The average Caesarean section deliveries rate stands at 4.5% with a disparity of 3.2% in rural and 9.3% in urban areas^14^. While many studies have focused on assessing the unmet obstetric needs and the causes of high MMR^15–18^, little is documented on the lessons learnt in the implementation of task sharing policy for reduction of maternal mortality in Tanzania. This study analysed lessons from the performance of Caesarean section by Assistant Medical Officers (AMOs) in Tanzania as part of the task sharing strategy.

### METHODS

**FIGURE 1.**
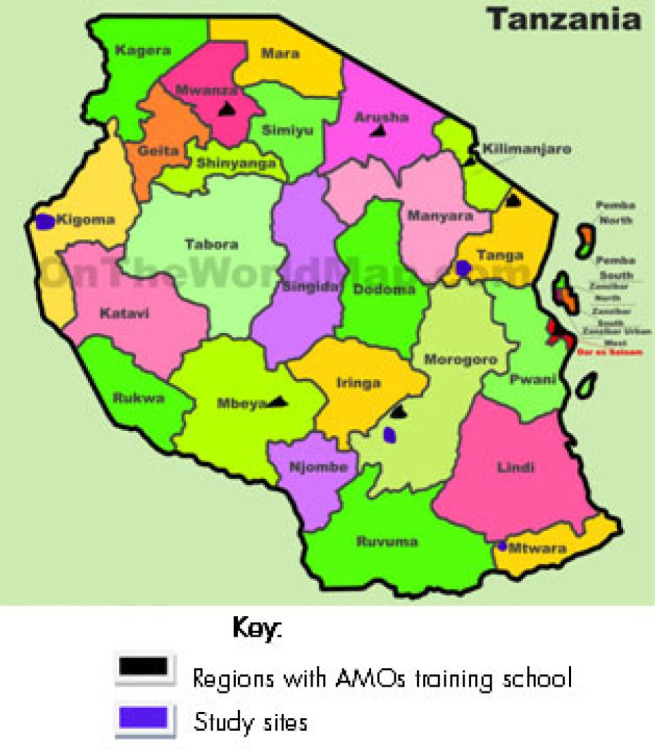
Study sites and AMO training schools in Tanzania

**Source:** Adapted and inked of a map from http://ontheworldmap.com/tanzania/tanzania-regions-map.html

An exploratory qualitative case study was conducted in 4 districts of Tanzania mainland from 4 regions that are located in 4 of the seven geographical zones of the country (table 1). These Districts are; Handeni from Tanga region in the Northern zone, Kilombero district from Morogoro in the Eastern zone, Masasi from Mtwara region in the Southern zone, and Kasulu from Kigoma region in the Western zone (fig 1). These regions were selected because of their variations in women's access to Caesarean Sections and their performance of Caesarean sections^10^. Specifically, data collection was conducted in 4 Districts hospitals and 2 upgraded health centres that were supported by the Word-Lung Foundation and 2 AMO schools between September 2015 to February 2016. Selection of health facilities considered the type of the health facility, type of ownership (Government or Faith-Based Organization ownership) and rural-urban dichotomy.

**TABLE 1:** Socio-demographic characteristics

Zone	Regions
Central zone	Dodoma and Singida
Eastern zone	Coast, Dar es Salaam and Morogoro
Lake zone	Kagera, Mara, Mwanza, Shinyanga, Simiyu and Geita
Northern zone	Arusha, Kilimanjaro, Manyara and Tanga
Southern zone	Lindi and Mtwara
Southern highlands	Iringa, Mbeya, Ruvuma, and Njombe
Western zone	Katavi, Kigoma, Rukwa and Tabora

#### Study Participants

This study involved AMOs practising at the 4 study districts, a retired AMO and AMO trainees from 2 purposefully selected AMO training schools. The practising AMOs were purposefully selected to include those in the management position at the health facility, those performing surgery and those who have served for more than 10 years. For those in the management position, we aimed at capturing their experiences on the availability of equipment and supplies, experience on the referral system and incentives to the AMOs for the performance of Caesarean section. For the practising AMOs, we aimed at capturing their experiences on the performance of the Caesarean section. For the retired AMO, we aimed at capturing the experience of the AMOs training as it has evolved with time. For the AMO trainees, we aimed at capturing their experience on the training of AMOs regarding the performance of the Caesarean section. The AMO trainees involved randomly selected participants to participate in a Focus Group Discussion (FGD) among those who were available during the days of this study. All the participants for the FGD were in the second year of training.

#### Data Collection

Ten (10) Key Informant Interviews (KIIs) were conducted with 9 practising AMOs and one retired AMO in the 4 zones. Each KII was carried out in the office of the key informant. The interview lasted between 50 and 100 minutes. Further, 4 FGDs with AMO trainees were conducted, 2 with females and 2 with males. 2 of the FGDs were conducted at Bombo AMO School and the other 2 at St. Francis Ifakara AMO School. The number of participants in each FGD ranged between 7 and 12. A total of 35 AMOs trainees participated in the FGDs. The FGDs lasted between 55 and 120 minutes. A researcher and a research assistant who assisted with note taking and recording of discussions moderated each FGD. Both the KIIs and FGDs were audio-recorded with permission of the participants.

The KIIs and FGDs were conducted by researchers with varying experience in conducting qualitative studies in maternal health ranging from 5 to 15 years. The research team included a nurse-midwife, a medical doctor with training in health systems and a sociologist. The researchers were assisted by trained experienced research assistants with a background in nursing and social sciences.

Semi-structured interview and FGD guides that were developed in collaboration with members from Averting Maternal Deaths and Disabilities from Columbia University, Traction, Eastern, Central and Southern Africa Health Community and the 4 country teams (Kenya, Tanzania, Uganda and Zambia) were used for data collection. These tools were guided by 8 thematic areas of; policy and regulations, human resources management, training, skill mix, referrals, stakeholders and professional associations and infrastructure, medical equipment and supplies. The tools were interpreted into Kiswahili language before being used.

#### Data Analysis

Interviews and FGDs transcripts were transcribed verbatim. The Kiswahili transcripts were then translated into English to ensure that data is accessible to non-Kiswahili speaking colleagues from TRAction. A codebook containing the domains under study that was developed before the data collection process was used to guide the initial coding process. From the study findings, the codebook was enriched and updated to include subdomains and updated domain definitions. The final codebook was structured into 24 domains that originated from the major 8 domains of this study. In the beginning, the research team read and re-read the transcripts to familiarise with the data before the coding process. The team met together where each one coded at least 2 transcripts and met together to discuss the codes and coding process for harmonisation or clarification and finally agreed on the final codes. 2 separate researchers coded at least one transcript. After agreeing on the codes and coding process, the team distributed the transcripts among each other for the coding process. All the coded transcripts were then organised by using NVIVO10 qualitative analysis software by QSR international.

From the reproduced reports from the NVIVO, the research team subdivided the domains to one another for analysis. Qualitative content analysis was used to guide the analysis. Codes were extracted from the reduced meaningful unit and wrote across the margins. Through comparison, checking, and rechecking of similarities and differences between the sub-categories, the sub-categories were sorted to form categories to reflect the manifest content of the text. The final codes and categories were agreed upon among the team and the findings were presented with the support of succinct quotes.

#### Ethical Considerations

Ethical approval was obtained from the Senate Research and Ethics Committee of the Muhimbili University of Health and Allied Health Sciences (Ref. No. 2015-06-09/AEC/Vol. IX/103). Permission to conduct the study in the 4 study settings was granted by the Ministry of Health, Community Development, Gender, Elderly and Children. Written informed consent was obtained from each participant after receiving explanations about the study aims and they were informed that their participation was purely voluntary and they were free to decline or withdraw at any time in the course of the study. Participants’ privacy was assured by not using their names during the data collection process and any of their identity in reports or publications. Special permission was requested for on the use of an audio recorder during interviews and discussions.

### RESULTS

From the analysis of the KIIs and FGDs 3 categories emerged; unfavourable working environment at the health facilities, poor incentive system and support towards improving the performance of Caesarean section by AMOs. The 3 categories (Table 2) were further grouped into 2 major themes; motivations for the performance of Caesarean section by AMOs and the de-motivations for Caesarean section performance.

**TABLE 2:** Summary of Findings

De-motivations for Caesarean section performance by AMOs	Motivations for the performance of Caesarean section by AMOs
Frustrating work environment	Support towards improving the performance of Caesar ean section by AMOs
Underequipped health facilities Limited capacity for effecting the referral of patients from the lower level to higher-level facilities Supervision limited to managerial instead of technical support The critical shortage of staff for skill mix	Recognition of the work done by AMOs by higher authorities and other stakeholders Contextualised incentives to AMOs in some places Stakeholders support to AMOs training, mentoring and improving the working environment
**Poor incentive system** Lack of clear career path for AMOs Variation in support for AMO training Overburdened AMOs in a situation of less compensation and often delayed	

#### Motivations for the Performance of Caesarean Section by AMOs

Informants revealed the existence of some motivations for the performance of the Caesarean section by the AMOs. These included; recognition of the work done by AMOs by higher authorities and other stakeholders, contextualised incentives to AMOs in some places and the stakeholders support to AMOs training, mentoring and improving the working environment for the performance of Caesarean section.

#### Recognition of the work done by the AMOs by Higher Authorities and Other Stakeholders

Recognition of the work done by AMOs by the ministry of Health and other higher government authorities was stated by the AMOs to motivate them to perform better. They added that the acceptability and continued engagement of their association that had been established in 2012 in matters pertaining delivery of health care services by the government and other stakeholders was a sign that they are recognised and supported.

*“…We work hand in hand with the ministry of health, we are invited in many meetings and conferences, and this was not there before…we are happy when we see that there are people out there in power who recognise our work…this makes us work more and more…” [KI-Kigoma]*

#### Contextualisation of incentives to the AMOs

In some places, informants stated that regardless of the financial crisis communicated by their immediate bosses, yet the councils had devised some top-up allowances in addition to the documented allowances. They mentioned some of the documented allowances to be on-call allowances and extra-duty allowances. They further added that the top-ups included; provision of soft loans for building houses to the AMOs and transport allowances. They stated that these financial incentives were motivating them to stay and to work hard as they felt that they were valued.

*“…I feel good to be here, although not every time I receive the top-up allowances, I can say most of the time I receive transport allowance. …. I am aware that in some neighbour districts, they receive also housing loan, it is a good thing. I heard some of our leaders here discussing the possibility of introducing housing loan as an incentive in our district as well… …this makes us feel valued and thus perform more….” [KI-Morogoro]*

#### Stakeholders’ support to AMOs training, mentoring and improving the working environment

From 2 regions out of the 4 regions under this study, we were informed that they were receiving the support from an NGO that was carrying out training on (CEmONC) to AMOs and nurses and facilitating post-training supportive supervision, the World Lung Foundation (WLF). They added that WLF carried supportive supervision to AMOs and other cadres involved in skill mix for emergency obstetrics care. In that system, WLF did; training, coaching, mentoring and provided equipment that were necessary for carrying out Caesarean section.

*“…One NGO [mention the name] come with their gynaecologists, anaesthetist and nurses, we work with them and they help us with the difficult cases that we had prepared because they always inform us before their arrival…”* [KIKigoma]

In some places the NGO supported the performance of Caesarean section through; provision of equipment and other medical supplies and improving the infrastructure like operating theatres. This support not only helped to improve the performance of the Caesarean section but also made the AMOs to be motivated in their work. Respondents expressed their satisfaction and gratitude to partners’ support in this activity.

*“…As I mentioned earlier, we are so grateful to [mention an NGO] for their support, they help us with medication, equipment every 3 months, and they do service for theatre equipment after every 3 months, thanks to their support we do not have a shortage of theatre equipment anymore except the drugs that are used and run out of stock…”* [KI-Morogoro].

#### De-motivations for Caesarean section performance by AMOs

From the interviews, informants stated the existence of demotivation that jeopardise Caesarean section performance. 2 categories emerged from participants’ narratives regarding de-motivations for Caesarean section performance; these were; frustrating work environment and poor incentive system.

#### Frustrating work environment

Participants reported frustrating work environment for the performance of Caesarean section. The frustrating work was attributed to; underequipped health facilities, limited capacity for effecting the referral of patients from lower-level to higher-level facilities, supervision limited to managerial instead of technical support and a critical shortage of staff for skill mix.

#### Under-equipped health facilities

Informants stated that most of the health facilities at the districts were grossly under-equipped to an extent that the effective performance of the Caesarean section was jeopardised. The health facilities suffered from; shortage of medical supplies, shortage of theatre equipment like cardiac monitors, dilapidated building, shortage of water supply and necessary drugs for carrying the Caesarean section. Absence of these necessities not only hijacked the performance of Caesarean section but also acted as a de-motivator to the AMOs in Caesarean section performance.

*“… There are shortages of equipment, instruments, and medicines are not enough. …. Some days we have anaesthesia drugs, and other days we do not. Especially these spinals (anaesthesia) are not enough, it causes problems. We don't have Anaesthesia machine, what we use is a small machine for blood pressure …”* [KI-Kigoma]

#### Limited capacity for effecting the referral of patients from the lower level to higher-level health facilities

Participants reported lack of ambulances at the health centres for effecting smooth referral of the patients. The reason of lack of ambulances varied across the 4 councils, in 2 councils, the vehicles were old and when taken for repair they were never sent back to the health centres but instead they were assigned to other activities at the council level. In the 3^rd^ council, the ambulance was there at the health centre, however, there was money to fuel it, while in the 4^th^ council, the vehicle was broken after an accident and there was yet not enough money to fix it. Participants added that lack of ambulances was a barrier for successful referral of women with complicated labour to the referral health facilities for comprehensive obstetric care.

*“…Previously every health centre had a vehicle and it was easy for patients around that area but it was taken by the district because there are other activities in the district and there is no vehicle there, so they decided to take those cars from health centres and assigned them to other activities… In this district council, there are 3 health centres. I do not think there is any car in any of these health centres …”* [KI-Mtwara].

In another place, participants reported lack of fuel as hindrance to ambulance operation and therefore women had to incur the cost of fuel for them to be transferred to the referral health facility for adequate obstetric management:

“*… Sometimes the ambulance has no fuel so we have to ask the women/relatives to pay for the fuel for them to be taken to the referred hospital…”* [KI-Tanga]

#### Supervision limited to managerial instead of technical support

Participants reported that during supportive supervision, they expected to also receive technical support that was lacking. The current routine supportive supervision by the council's health management teams was primarily focusing on managerial issues. For the AMOs the technical competencies were important for them to effectively perform the Caesarean section rather than the managerial competencies.

“…*they come to observe our performance and identify our problems but they don't work with us unlike the (mention an NGO) people who come with their gynaecologists, anaesthetist and nurses, we work with them and they help us with the difficult cases ….”* [KI-Morogoro]

#### Critical shortage of other health workers needed for caesarean section delivery

From all the 4 study districts, informants stated the existence of a critical shortage of personnel who work hand in hand with AMOs in Caesarean section delivery. In 3 out of the districts, anaesthetists were not available and their roles were delegated to medical attendants or clinical officers who received a 4 months’ short course on anaesthesia. In some places, there was a critical shortage to an extent that laboratory technicians were opted as assistant surgeons during caesarean section delivery.

*“… You need at least 4 people for a Caesarean section. However, here we have a serious shortage; sometimes we even use people from the laboratory as assistant surgeons or circulating nurse. I am the only surgeon and have only one anaesthetist and she is my assistant surgeon…”* [KI-Kigoma]

#### Poor Incentive System

Participants reported the existence of varying incentives from some incentives to lack any form of incentive across the 4 districts. The informants revealed; absence of clear career path for AMO cadre; varied financial support to the AMO trainees, lack of scholarships and limited financial incentives for AMOs.

#### Lack of clear career path for the AMOs

Informants of this study stated that becoming an AMO was like going to the end of one's career. Although some universities admitted AMOs for the Doctor of Medicine degree, participants stated that it was very hard for the AMOs to survive the competition with fresh graduates from high schools. They further added that Medical schools provided very few slots for the AMOs and other equally qualified clinical officers to compete for admission to the doctor of medicine programme.

*“… Imagine, you trained for 3 years to become a clinical officer, you worked for at least 3 years before joining AMO program which is 2 years… You need 5 years to become a medical doctor. That is like saying you need 13 years to acquire a degree. That not being enough, to be admitted, you are subjected to stiff competition with fresh graduates. … Indeed, becoming an AMO is like a dead end in your career…”* [KI-Tanga]

#### Variation in support for AMO Training

Focused group discussion with AMOs trainees revealed that there was a big variation in financial support to the training of AMO students across the districts. While some districts fully supported their Clinical Officers to join the training, some did not support at all. Some FGD members reported having received partial support (covering full tuition fees and stationeries, or half tuition fees and full stationeries or tuition fees without stationeries or stationeries without tuition fees). Another group reported having received full support (full tuition fees and stationeries) and the last group reported to have not received any kind of support from their districts.

*“…When we were about to go for studies, our employer told us that it was upon ourselves to pay for our studies or not to join the studies. That has been the situation to now. I and colleague from the same district we pay for our studies.…”* [FGD Member no.10 Male group]

Another FGD member lamented that regardless of the promise of support, that support was never provided.

*“… I am getting just words of support from my bosses that I will be given stationeries, but since when I came here now is my final year, I have not seen that support for stationeries…”* [FGD Member no. 2 Male group]

For some AMO trainees who were not receiving any form of financial support from their districts, they complained that even moral support was not there. They added that it was difficult for them to leave their workplace for AMO training.

*“… I am giving an example from my own experience because I was harassed very much. There was a lot of disturbance when I applied for studies, I was told that I do not qualify and after all, the cadre is not needed. This happened while I had already got my admission letter…it took much effort until I was released to come here to study without a single penny of support…”* [FGD Member number 1, Female Group]

#### Overburdened AMOs in a situation of less and often delayed compensation

From interviews, the AMOs explained that there was reported limited financial capacity at the districts. They added that the latter resulted into; failure of some districts to provide financial incentives or delayed payment of the financial incentives. Furthermore, the AMOs reported that with the severe shortage of health workers, they had to work for extended hours to ensure that services are not interrupted. However, they complained that regardless of this labouring, they were not compensated accordingly, and even the little compensation sometimes was seriously delayed.

” …*. there has also been a problem with motivation to work for example; there are few workers and sometimes they are required to work extra hours but the extra hours’ allowances are not given accordingly…for instance now, the last allowances were paid 3 months ago…”* [KI-Kigoma]

## DISCUSSION

We aimed to analyse lessons from the performance of Caesarean section by Assistant Medical Officers (AMOs) in Tanzania as part of the task sharing strategy. From the findings of this case study, we have found that the performance of the Caesarean section by the AMOs is motivated by the support from various stakeholders towards improving the performance of Caesarean section by the AMOs. Frustrating work environment and poor incentive system were found to be the demotivators for the performance of caesarean section by the AMOs.

The motivations revealed by this study are similar to what is documented by other studies with regards to the motivation of health workers in Tanzania^19,20^. Recognition of the performance of the AMOs as found in this study is in line with prominent human resources motivation theories^21–23^. The latter reveals that when an effort is recognised, it leads to more efforts that result into increased performance. The AMO cadre since its introduction has been the backbone of health care service provision at the district level where there is a critical shortage of medical doctors^24^. Recognising their efforts by higher authorities as revealed by this study is in line with the Vroom's expectancy theory of motivation^21^. According to Vroom, recognising the efforts of the workers tend to increase their performance. The authors feel that by recognising not only the contribution by AMOs but of all the workers in the health sector may have positive outcomes in reducing the maternal mortality in this country that are still unacceptably high^10^.

Provision of housing or housing allowances and or in this case loans for building houses is retention strategy used in many parts of the world for retaining health workers in rural and remote areas^25–27^. Although the latter strategy was not found to be universally implemented in all the study districts, our findings have revealed that in the places where it was implemented, AMOs were motivated. Motivation is closely linked to the retention of health workers^28^. The reduction of maternal mortality among other things requires the retention of AMOs and other cadres required for skill mix in the district health system. Performance of Caesarean section delivery is a product of teamwork that requires the availability of a surgeon (in this case, the AMO), a nurse, a midwife, an anaesthetist, an assistant surgeon and a lab technician. Existence of the stakeholders on improving the performance of Caesarean section conform to what was documented by the global health initiative as a shared responsibility in ensuring the availability of adequate health workforce^29^. Stakeholders working together in improving the performance of the health workforce is a good sign towards the sustainability of the task sharing strategy in Tanzania. However, our findings point out that the donor-driven support through the NGOs as found in this study contradicts the aim of the government of reducing donor dependency syndrome in the health sector^30^.

Frustrating work environment as revealed in this study is not unique to the AMOs in Tanzania. It is a crosscutting problem in the health sector in Tanzania as pointed out by other studies on health workforce retention in rural districts of Tanzania^31,32^. The latter stated that the working environment at the district health system was poor to an extent that health workers and health managers felt as if the government forgot them and were looking for opportunities to leave the districts.

Under-equipped facilities as found in this study is not facing AMOs in solitary. The latter situation equally challenges all other cadres at the district level and below. The situation revealed is similar to what Mbaruku et al ^33^ documented in a countrywide survey on health workers’ dissatisfaction on working environment. In the latter study, Mbaruku reported that shortage of drugs and supplies was the most dissatisfying aspect of the work environment and it contributed to demotivation of the health workers.

Critical shortage of health workers for skills mix in Caesarean section delivery as revealed in our study, is not a new phenomenon and is not unique to the Caesarean section delivery, rather it is a chronic problem in the health care services provision arena in the health sector in Tanzania^34^. As for other parts of the world^27^ the rural areas are the most affected places^35–37^. Shortage of health workers is recognised at global level as among the main challenges rendering under-performance of many health systems^38^. In sub-Saharan Africa where 66% of maternal deaths occur annually, at least one million new health workers are needed to rescue the current situation^39^. In Tanzania where the health system operates with less than 50% of the required health workforce, raising performance of the available overstretched health workforce is perhaps the immediate solution at hand as training and deploying new cadres is time consuming and costly.

The poor supervision system as revealed by our study is contrary to what the government advocates for on reduction of maternal mortality in Tanzania^11,12^. The government recognises that without adequate supervision the current MMR will not go down. Our findings are also in line with findings by study carried out in Ghana which indicated that good supervision was associated with improved maternal health outcomes^40^.

Our study revealed limited career path, limited scholarships for AMOs training and limited financial incentives in overwhelmed burden of work as among the demotivators to the AMOs. The findings are similar to what other studies found in Tanzania with regards to the incentives in health workers performance^34,41–45^.

Although offering career opportunity is well recognised by the ministry of health of Tanzania as an important non-financial incentive for motivating health workers especially in rural areas to stay and perform well, its implementation is challenged by scarcity of resources^34^. Experiences from South Africa revealed that offering career opportunities not only motivate health workers but also retain them in working places^46,47^. The scarcity of resources for offering financial incentives as documented in our findings is similar to what other studies documented in Tanzania^20,45,48^. The latter is similar to what Dambisya documented in a study in the Eastern, Central and Southern Africa. In the latter study, Dambisya stated that many countries of Eastern, Central and Southern Africa have many written good financial incentives but its implementation is jeopardised by their limited financial resources^49^.

### Methodological Consideration

We discussed the methodological considerations by first discussing the trustworthiness of the findings and then the study limitations. Trustworthiness is attained in a qualitative study when the findings of such a study are worth believing^50^. Four (4) criteria are used to assess the trustworthiness of a qualitative study; credibility, dependability, transferability, and confirmability^51^. The credibility of the findings of this study was enhanced through the triangulation of informants with experiences and rich information on the study questions. In order to enhance the credibility and dependability of this study, we used triangulation of data collection techniques, study settings, and researchers. Data were collected using interview guides and a focus group discussion guide in 4 different zones with different cultural and socio-economic activities. In order to confirm that the findings reflected informants’ perspectives rather than the researchers’ understanding of the question under study, categories were inductively generated using content analysis and presented with the support of sub-categories and quotes. The transferability of the findings of this study is enhanced through the description of the study setting, context, data collection process, and analysis.

The findings of this study are subject to the following limitations. First, the fact that health workers (medical doctor, midwife) were involved in conducting the interviews, might have introduced social desirability from the participants. However, the triangulation of informants, setting and having research assistants with social sciences background offset this limitation. Second, only 4 districts were involved and in a few selected facilities, this may have left out other variations of experience from the remaining part of the country uncovered. However, the involvement of the different categories of informants add richness to this study and enhance the applicability and the truth value of these findings. Third and last, this was a qualitative study and thus only a few selected informants were interviewed. The latter has the magnitude of motivations and de-motivations uncovered. However, this study provides insights into the situation at the districts and may set for a bigger study to address the health workforce performance challenges at the districts. Involvement of the terrain from the district hospital to a dispensary offset the latter limitation. It should be noted that the findings of this study reflect the situation during the period when data collection for this study was done.

## CONCLUSION

Our findings underscore that improving the performance of Caesarean section delivery by the AMOs as a task sharing strategy is surrounded by motivators and de-motivators which are linked to their training and workplace. Improving work environment and appropriate incentives, both financial and non-financial are necessary for addressing the demotivators for the performance of Caesarean section delivery by the AMOs. Joint stakeholders’ efforts are key in the realisation of the latter as massive investments are needed. For smooth implementation of task sharing and thus help in reducing the maternal mortality, measures must be instituted to ensure availability of all necessities.
